# Subtype-Specific Vulnerability of Spiral Ganglion Neurons in Sensorineural Hearing Loss Across the Lifespan

**DOI:** 10.3390/brainsci16060572

**Published:** 2026-05-28

**Authors:** Yuanyuan Peng, Qingchen Wang, Shuyao Qiu, Haichang Diao, Tingting Liu

**Affiliations:** 1 School of Public Health, Southeast University, Nanjing 210009, China; 220234062@seu.edu.cn (Y.P.); 220234006@seu.edu.cn (S.Q.); 220234047@seu.edu.cn (H.D.); 2 Institute of Reproduction and Development, Fudan University, Shanghai 200030, China; wangqc@fudan.edu.cn

**Keywords:** sensorineural hearing loss, spiral ganglion neurons, neuronal heterogeneity, subtype-specific vulnerability, auditory neuroscience, aging, neuroprotection

## Abstract

**Highlights:**

**What are the main findings?**

- Spiral ganglion neuron (SGN) degeneration in sensorineural hearing loss is not uniform, but is shaped by the interaction between subtype identity, life stage, and injury context.
- Evidence from mature and aging cochleae suggests that Ic- or LSR-related SGN populations are often among the most vulnerable, whereas developmental evidence is better interpreted as disruption of subtype specification and synaptogenesis.

**What are the implications of the main findings?**

- A lifespan-based and subtype-informed framework may improve the current understanding of cochlear neuropathy, age-related auditory decline, and the neural basis of impaired hearing in complex listening conditions.
- Future therapies for sensorineural hearing loss should move beyond generalized cochlear protection toward stage-aware, mechanism-based, and potentially subtype-targeted strategies for SGN preservation and repair.

**Abstract:**

**Background:** Sensorineural hearing loss (SNHL) is increasingly recognized as a disorder involving not only hair-cell damage but also selective degeneration of spiral ganglion neurons (SGNs). Recent single-cell, molecular, and functional studies have refined the classical type I/type II classification of SGNs by identifying distinct Ia, Ib, and Ic subtypes within type I neurons. This review aims to synthesize current evidence on how SGN vulnerability is shaped by the interaction between subtype identity, life stage, and injury context. **Methods:** We conducted a critical narrative review of recent studies on SGN heterogeneity and subtype-specific vulnerability across development, maturity, and aging, with particular attention to molecular profiling, functional studies, and emerging therapeutic strategies. **Results:** SGN degeneration in SNHL is not uniform. During development, the available evidence mainly supports the vulnerability of subtype specification, synaptogenesis, and activity-dependent maturation, rather than direct selective degeneration of mature Ia/Ib/Ic identities. In the mature cochlea, subtype-specific differences in synaptic architecture, ion-channel composition, and metabolic demand appear to shape responses to noise, ototoxic drugs, and ischemic stress, with Ic-related populations often showing greater vulnerability. During aging, cumulative mitochondrial dysfunction, oxidative stress, chronic inflammation, and declining neurotrophic support may progressively unmask differences in subtype resilience and contribute to age-related auditory decline. **Conclusions:** A lifespan-oriented and subtype-informed framework may improve the current understanding of selective SGN degeneration and support the development of more precise neuroprotective and reparative strategies for SNHL.

## 1. Introduction

Hearing loss is a major global health problem that significantly affects communication, mental health, and social participation. According to WHO projections, by 2050, nearly 2.5 billion individuals worldwide will have some degree of hearing impairment, with more than 700 million expected to require hearing rehabilitation [1,2,3]. SNHL is the most prevalent form of hearing impairment [4] and is primarily associated with dysfunction of cochlear hair cells (HCs) and spiral ganglion neurons (SGNs), the primary afferent neurons responsible for transmitting auditory signals from the cochlea to the brainstem [2,5,6,7,8]. SGNs play a critical role in auditory signal transmission and hearing perception. In mammals, mature SGNs have very limited regenerative capacity [9], making them particularly vulnerable to various insults, including noise overexposure [10,11], ototoxic drugs [12,13,14], genetic mutations [15,16], aging [17,18], and inflammation [19,20]. Damage to SGNs can therefore lead to irreversible neural degeneration and permanent hearing deficits [21].

Traditionally, SGNs are categorized based on their morphology and connectivity into myelinated type I neurons (constituting approximately 95%) and unmyelinated type II neurons (accounting for about 5%) [22,23]. More recently, studies based on single-cell transcriptomic analyses have established that spiral ganglion neurons (SGNs) comprise molecularly and functionally distinct subtypes [24,25,26,27].

Recent work has made it increasingly clear that SGN degeneration in SNHL is not a homogeneous process. Instead, subtype identity, developmental timing, and injury context appear to interact in shaping selective vulnerability. Although studies of SGN heterogeneity, cochlear synaptopathy, and auditory aging have expanded rapidly, these findings remain dispersed across developmental biology, auditory neuroscience, and regenerative hearing research. A critical synthesis focused specifically on subtype-dependent SGN vulnerability across the lifespan is still lacking. In this review, we examine how SGN subtype identity influences susceptibility during development, maturity, and aging, and we discuss how this framework may refine current concepts of neuroprotection, repair, and precision intervention in SNHL.

## 2. The Cochlea, Spiral Ganglion Neurons, and the Clinical Context of SNHL

The cochlea is the sensory organ responsible for converting sound-induced mechanical vibrations into electrical signals that can be processed by the central auditory system [28]. Within the cochlea, hair cells act as mechanosensory receptors that transduce basilar membrane motion into neural signals [29,30,31]. These signals are then conveyed to the brainstem by spiral ganglion neurons (SGNs), which constitute the primary auditory afferents and form the auditory nerve.

Damage to either hair cells or SGNs can cause sensorineural hearing loss (SNHL). Although hair-cell pathology has traditionally received greater attention, growing evidence indicates that SGN degeneration is also a major determinant of hearing impairment, particularly in conditions involving synaptic loss, neural degeneration, or reduced responsiveness to auditory rehabilitation. Preservation of SGNs is therefore essential not only for maintaining auditory nerve function but also for optimizing the outcomes of hearing restoration strategies such as cochlear implantation.

SNHL refers to hearing impairment caused by dysfunction of the inner ear, the auditory nerve or their central connections [32]. It is the most common form of hearing loss, accounting for approximately 90% of all hearing impairments [33]. SNHL is primarily caused by irreversible damage to cochlear hair cells or by degeneration and loss of SGNs [9,34,35,36]. Because SGNs are highly differentiated neurons with limited regenerative capacity, injury caused by aging, noise exposure, ototoxic drugs, or genetic defects is often permanent [21,37,38,39,40,41]. Current treatment options remain limited and are largely restricted to hearing aids and cochlear implants (CIs) [42,43]. Importantly, CI performance depends strongly on the survival and functional integrity of residual SGNs [44], which makes SGN protection and regeneration central goals in contemporary hearing-loss research [9,45,46,47,48].

## 3. Revisiting SGN Heterogeneity: From Classical Morphology to Molecular Subtypes

SGNs are the primary afferent neurons of the peripheral auditory system and relay electrical signals from inner hair cells (IHCs) to central auditory nuclei [9,49]. Their structural, molecular, and functional diversity is fundamental to normal sound encoding and is increasingly recognized as a key determinant of vulnerability in SNHL. Classical anatomical studies provided the first broad classification of SGNs, whereas electrophysiology, molecular profiling, and single-cell omics have since revealed substantially greater heterogeneity. This shift from a binary anatomical classification to a subtype-resolved framework is critical for understanding why SGN degeneration is selective rather than uniform.

### 3.1. Anatomical and Morphological Organization

Anatomically, SGNs display substantial heterogeneity in soma size, shape, innervation pattern, and spatial distribution. Based on morphology and peripheral targets, SGNs are classically divided into two major types—type I and type II neurons. Type I SGNs account for roughly 95% of the total population [50] and are typically myelinated bipolar neurons with relatively large somata [51]. Under in vitro culture conditions, they may also exhibit unipolar morphology and the balance between bipolar and unipolar forms can be influenced by factors present in the culture environment [52]. Each type I SGN forms a single peripheral synapse with one IHC and constitutes the principal neural pathway that transmits cochlear information to the central auditory system [51].

In contrast, type II SGNs are much less numerous, comprising approximately 5–10% of the SGN population [50]. These neurons are unmyelinated or only minimally myelinated, have smaller somata, and extend peripheral fibers to multiple outer hair cells (OHCs). Their precise functions remain incompletely understood, but current evidence suggests roles in cochlear damage signaling, modulation of cochlear mechanics, and adaptive responses to injury [53,54].

**Figure 1:**
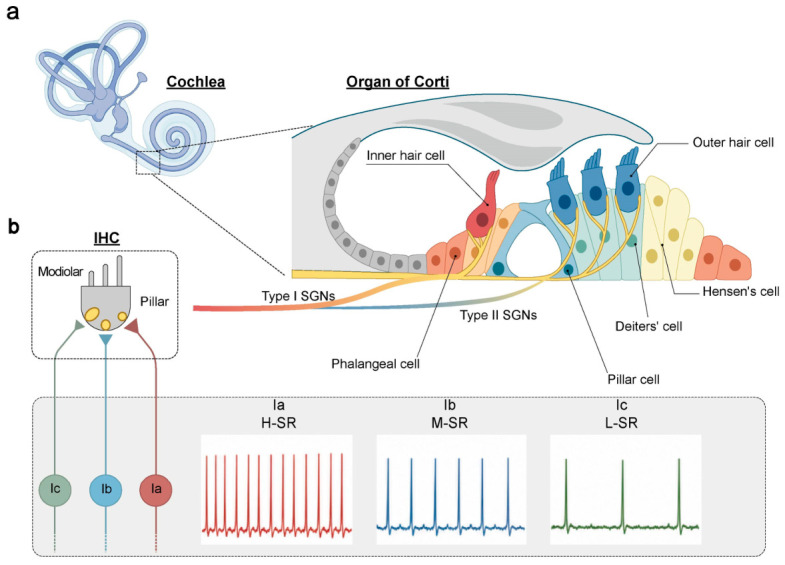
Cochlear structure and afferent innervation patterns of spiral ganglion neurons. (**a**) Schematic overview of the cochlear sensory epithelium showing major cell types, including outer hair cells (OHCs), inner hair cells (IHCs), type I and type II spiral ganglion neurons (SGNs), pillar cells, phalangeal cells, Deiters’ cells, and Hensen’s cells. (**b**) Schematic representation of type I SGN subtype organization and qualitative firing-pattern differences adapted from Petitpré et al. [56]. Type I SGNs can be subdivided into Ia, Ib, and Ic populations, which approximately correspond to high-spontaneous-rate (HSR), medium-spontaneous-rate (MSR), and low-spontaneous-rate (LSR) fibers, respectively, and differ in functional properties and preferred innervation patterns at IHC synapses. The traces are not original single-unit recordings and are intended for conceptual illustration only; they should not be interpreted as quantitative electrophysiological data. Created in BioRender.

In addition to this major type I/type II division, SGNs also exhibit spatial gradients along the cochlear spiral. Soma size and axon growth characteristics are not uniformly distributed, but instead vary tonotopically along the apical-to-basal axis [55]. Because these gradients interact with subtype distribution, tonotopic organization, and vulnerability, they are discussed separately in Section 3.3. Figure 1 summarizes the cochlear structure and afferent fiber innervation patterns.

### 3.2. Functional Specialization and Subtype-Defining Molecular Signatures

Beyond gross morphology, SGNs also exhibit marked functional and molecular heterogeneity. This diversity enables the auditory system to encode sound intensity, temporal structure, and complex acoustic environments with high precision [57,58]. Because natural sounds span a wide dynamic range, distinct SGN populations provide complementary sensitivities and response ranges, thereby preserving auditory performance under changing acoustic conditions. This functional diversity depends on the accurate synaptic organization between IHCs and SGNs [59]. Two distinct but complementary mechanisms appear to contribute to SGN subtype diversification. First, molecular regulation of postsynaptic architecture is essential, disruption of the synaptic adhesion molecule RTN4RL2 alters IHC synaptic structure, reduces postsynaptic density (PSD) size, mislocalizes AMPA receptor components, and elevates hearing thresholds, illustrating how specific molecular shape SGN subtype function [59]. Second, presynaptic heterogeneity can emerge even in the absence of neurotransmitter release, suggesting that at least part of SGN diversification is established through developmentally programmed mechanisms that precede mature synaptic transmission [57,60]. Together, these postsynaptic and presynaptic determinants support the precise encoding of auditory information across diverse acoustic conditions.

Functionally, type I SGNs have long been categorized according to spontaneous firing rate (SR) as high-SR (HSR), medium-SR (MSR), and low-SR (LSR) fibers [51,61]. These groups differ in threshold, dynamic range, and likely contribution to auditory coding. HSR fibers have low sound thresholds and are optimized for detecting faint sounds in quiet conditions [62,63]. In contrast, LSR fibers have higher thresholds and broader dynamic ranges, making them particularly important for encoding suprathreshold sounds and listening in noisy environments [27,57,60]. Although some studies suggest relative tolerance of LSR fibers to certain acute insults, these populations appear disproportionately affected in chronic degenerative states, including age-related hearing loss [18,64]. This apparent paradox highlights the need to distinguish short-term resilience from long-term vulnerability.

Single-cell transcriptomics studies have extended these functional categories into a more stable molecular framework. Several key studies have identified three major molecular subtypes within type I SGNs, designated Ia, Ib, and Ic [24,25,65]. These subtypes are widely considered to approximate HSR-, MSR-, and LSR-like populations, respectively, linking stable molecular identity to functional firing properties [57,58]. Functionally, Ic neurons are thought to contribute disproportionately to sound encoding under noisy or high-intensity conditions, whereas Ia neurons are better suited for detecting weak sounds in quiet environments [27,57,58,60,62]. Type Ib neurons appear to occupy an intermediate functional position.

These functional distinctions are accompanied by distinct patterns of peripheral innervation and molecular marker expression. In the primate cochlea, type Ia fibers preferentially innervate the pillar side of IHCs, type Ic fibers occupy the modiolar side, and type Ib fibers occupy intermediate positions [60,66]. Molecularly, Ia neurons are enriched in CALB2 and express genes such as Cacna1b, Cacna1h, and Cacna2d1 [24]; Ib neurons are characterized by CALB1 expression and regulation by Runx1; and Ic neurons are associated with POU4F1 and Mafb and display ion-channel expression profiles, including Kv1 and HCN channels, consistent with high-threshold, broad-dynamic-range firing properties [67,68,69,70,71,72,73,74]. A Grm8 reporter model has further supported Grm8 as a selective marker of Ic neurons and demonstrated its enrichment in the basal, high-frequency cochlea [69].

Taken together, these observations indicate that SGN heterogeneity is not simply descriptive, but mechanistically relevant. Molecular identity, firing properties, synaptic organization, and cochlear position are tightly linked, and this integrated subtype architecture likely underlies the unequal susceptibility of SGNs to developmental disruption, acoustic injury, ototoxicity, and aging. In this sense, subtype identity provides the conceptual bridge between SGN diversity and selective vulnerability.

### 3.3. Tonotopic Organization, Species Differences, and Limits of Subtype Extrapolation

Subtype identity should be interpreted in relation to cochlear tonotopy, because SGNs are not uniformly organized along the apical-to-basal axis. Classical anatomical studies have shown that SGN soma size and axonal growth properties vary according to cochlear position, with larger SGN somata generally observed in basal, high-frequency regions than in apical, low-frequency regions. Co-culture studies further suggest that the organ of Corti can influence SGN soma size, indicating that regional cochlear environments may contribute to anatomical SGN heterogeneity [55]. These anatomical gradients likely provide a structural basis for functional specialization and may also influence differential vulnerability under pathological conditions.

Subtype-resolved studies indicate that molecularly defined SGN populations are unevenly distributed along the cochlear spiral. In mice, calretinin-positive and calbindin-positive populations, corresponding broadly to Ia- and Ib-related groups, are more prevalent toward the apical cochlea, whereas CR-negative/CB-negative Ic-related neurons show the opposite pattern and are relatively enriched toward the basal cochlea [27]. Aging further alters this distribution, with preferential loss of Ic-related neurons reported particularly in middle and basal regions [27]. These findings indicate that subtype identity and cochlear location are interacting but non-equivalent axes of vulnerability.

This distinction is important when interpreting noise- and age-related studies. Evidence from molecularly defined mouse models supports differential synaptopathy among SGN subtypes, with Ic- or LSR-related synapses often showing greater vulnerability and Ia-related synapses showing relative resistance [26]. However, a finding obtained at a basal high-frequency region, such as the 32 kHz region, should not be generalized to mean that the basal cochlea is universally more vulnerable than all other regions. Rather, the available data support the more cautious conclusion that subtype composition, local tonotopic position, and injury paradigm jointly influence the observed pattern of damage [26,27].

Species differences represent an additional limitation when extrapolating SGN subtype frameworks across mammals. Much of the current Ia/Ib/Ic classification is derived from rodent studies, particularly in mice. However, SGN subtype proportions, marker expression, cochlear frequency range, spontaneous-rate distributions, and aging trajectories may differ across species. In gerbils, calretinin and Lypd1 expression have been used to distinguish H/M-SR and L-SR type I SGNs along the cochlear spiral, with Lypd1-positive, Ic-like neurons showing a decreasing gradient from base to apex [56]. This finding supports the possibility that tonotopic variation in SGN subtype distribution extends beyond the mouse cochlea. Functional studies of the gerbil auditory nerve are broadly consistent with this interpretation, showing that low-SR fibers have higher thresholds and are non-uniformly represented along the tonotopic axis, with low-SR-related responses being particularly relevant in higher-frequency cochlear regions [75]. Thus, molecular and physiological categories should not be assumed to map perfectly across species.

Primate and non-traditional mammalian models further highlight this caveat. In the common marmoset, type I SGN subgroups show partial conservation of early subtype-specification programs but also display species-specific differences in later marker expression [66]. Emerging single-cell studies in bat cochlea have identified species-specialized cochlear cell diversity and possible novel SGN populations, further emphasizing that subtype homology across mammals requires direct validation [76]. Therefore, extrapolation from mouse Ia/Ib/Ic populations to humans should remain cautious until subtype markers, spatial distributions, and functional correspondences are validated in primate and human tissues.

## 4. A Lifespan Framework for Selective Vulnerability

As terminally differentiated neurons, SGNs have limited regenerative capacity after injury [9,34,35,36], making their degeneration a major determinant of irreversible hearing impairment. However, SGN degeneration should not be regarded as a uniform endpoint. Instead, available evidence suggests that subtype identity interacts with developmental stage and injury context to shape selective vulnerability. A lifespan framework is therefore useful not only for organizing existing evidence, but also for clarifying when subtype-specific vulnerability is established, when it becomes functionally consequential, and how it may be therapeutically targeted. In the following sections, we examine how this interaction unfolds during development, maturity, and aging, and summarize the corresponding vulnerability patterns under major pathological conditions (Table 1).

### 4.1. Development: Vulnerability During Subtype Specification and Synaptogenesis

During development, SGN vulnerability is shaped less by mature physiological specialization and more by the ongoing establishment of subtype identity, synaptic connectivity, and activity-dependent maturation. At this stage, genetic and environmental perturbations may not simply injure SGNs, but may alter the developmental programs through which later subtype-specific function and resilience are acquired. This distinction is important, because developmental insults may have lasting consequences for SGN identity, connectivity, and function even when overt neuronal loss is not immediately apparent.

The critical developmental window for human SGNs spans the mid-to-late embryonic period through the perinatal stage [113,114]. Anatomical studies indicate that auditory neurons begin central migration at approximately gestational week 8 (GW08), with the early spiral structure formed by GW10 [114]. At the molecular level, dynamic changes in neurotrophin signaling occur around GW18–19. For example, the downregulation of BDNF/NT-3 together with the compensatory upregulation of NT-4 appears to mark the near completion of the primary developmental program in SGNs [114]. Perturbations during this period may therefore produce long-lasting and irreversible consequences for SGN number, connectivity, and functional maturation. Clinically, this developmental susceptibility is reflected by the increased ototoxic risk seen in preterm infants and neonates [83]. For instance, gentamicin-associated ototoxicity in neonates has been estimated at approximately 3% [84,85], and kanamycin can directly damage neonatal SGNs while sparing adult SGNs under comparable conditions [86,115,116]. Reduced drug clearance in the neonatal period may further increase the risk of inner ear accumulation and toxicity [87]. Similarly, young children show heightened susceptibility to cisplatin ototoxicity, with major downstream implications for language acquisition and psychosocial development [90,91].

Mouse models have been particularly informative because they reproduce key temporal features of human auditory development while allowing mechanistic interrogation. In mice, neuroblasts delaminating from the otocyst at E11.5 initiate auditory lineage differentiation [117,118]. Between E14.5 and E18.5, the auditory pathway establishes early axonal projections and initial peripheral–central connectivity, while subtype-specific molecular signatures can already be detected from E16 onward and continue to consolidate throughout the first postnatal week [68,88,119]. Spontaneous electrical activity peaks around postnatal day 7 (P7), the ear canal opens between P12 and P14 [119,120], and by P20 the peripheral auditory system is largely mature [121]. The auditory cortex critical period closes progressively around P28, accompanied by reduced plasticity [122]. Consistent with this timeline, single-cell transcriptomic and lineage-tracing studies suggest that molecular diversity and subtype specialization are largely established at birth and are subsequently consolidated during early postnatal development [68,69,70,71]. Gata3 acts early in embryogenesis by regulating Fgf10 and thereby helps establish conditions for later SGN differentiation [123], whereas Neurod1 appears to contribute to developmental programs linked to type Ic specification [118,124]. Comparative single-cell RNA sequencing between neonatal and adult cochlea further supports the conclusion that much of SGN molecular diversity is already present at birth [65]. Additional lineage-tracing work using Prph-p-mCherry models has shown that the proportion of labeled neurons in the adult cochlear hook declines from approximately 92% at P1 to 42% in adulthood, supporting the idea that type I SGN subtype composition is progressively refined during development rather than being fully stabilized at the earliest postnatal stages [69].

Genetic and environmental perturbations during this period can disrupt these developmental processes in several ways. Many hereditary forms of SNHL initially affect hair-cell development or function and subsequently lead to secondary SGN degeneration [101,102]. For example, *PCDH15* mutations have been identified in humans with hereditary hearing loss and can modify the phenotypic severity of co-occurring cochlear genetic defects [103]. Experimental evidence in mice further demonstrates that Pcdh15-null mutations abolish hair-cell mechanotransduction, disrupt SGN spontaneous activity, and impair Ia/Ib/Ic subtype specification [25]. Similarly, loss of Vglut3 impairs glutamatergic IHC-SGN signaling and perturbs activity-dependent subtype maturation [104]. Ototoxic agents, particularly aminoglycosides and platinum-based drugs [12,125,126,127,128], induce massive ROS production and oxidative stress within the inner ear, and neonatal SGNs appear especially sensitive to such injury [12,86,115,116,125,129,130,131]. Inflammation may also contribute, either directly or as a secondary response to cochlear damage. Upregulation of inflammatory genes has been observed in deafened rats [132], and CMV-induced cochlear inflammation in newborn mice can cause SGN loss and synaptic degeneration even in the absence of overt hair-cell destruction [133]. Nevertheless, direct evidence that inflammation preferentially affects specific SGN subtypes remains limited.

More broadly, this developmental context also requires a different interpretation of subtype-specific vulnerability from that applied to the mature or aging cochlea. Because Ia/Ib/Ic identities are still being specified and consolidated [68,69,70,71], developmental insults cannot be assumed to selectively eliminate fully stabilized neuronal subtypes. Rather, they may disrupt the developmental trajectories through which subtype identity, synaptic position, and activity-dependent maturation are acquired [25,104]. Direct evidence for differential degeneration among mature Ia/Ib/Ic populations during development is currently lacking [26,27]. Thus, during development, SGN vulnerability is better understood as disruption of subtype specification and synaptogenesis than as selective degeneration of fully stabilized Ia/Ib/Ic identities.

### 4.2. Maturity: Context-Dependent Injury Responses in Established Subtypes

Once SGNs reach functional maturity, subtype-specific physiological and metabolic properties become more stable. Accordingly, selective vulnerability in the mature cochlea appears to reflect the interaction between intrinsic subtype features and the specific nature of the insult, including acoustic overstimulation, ototoxic exposure, and ischemic stress [134,135]. This stage is therefore particularly informative for testing whether subtype identity translates into reproducible differences in injury susceptibility.

A further distinction is needed between cochlear synaptopathy and SGN neurodegeneration. Synaptopathy refers primarily to the loss or dysfunction of IHC-SGN ribbon synapses or peripheral terminals, often while SGN somata remain present. This process can reduce auditory nerve output and impair suprathreshold hearing without necessarily producing immediate neuronal death. By contrast, neurodegeneration refers to irreversible structural loss of SGN cell bodies or axons, which is more likely to occur after severe injury, prolonged trophic deprivation, or advanced aging [18,136]. This distinction is particularly important for subtype vulnerability, because Ic- or LSR-associated populations may show early synaptic vulnerability after noise exposure, whereas overt neuronal loss may appear later or under more severe degenerative conditions [26,77].

Noise exposure provides the clearest current example of mature subtype-selective vulnerability. Type Ia SGNs appear relatively stable under acoustic overstimulation, likely because of their larger PSDs and more robust synaptic microstructure [24,25,68]. Conversely, Ib/Ic- and especially Ic/LSR-associated synapses appear more susceptible in several noise- and age-related models [77,78,79]. This vulnerability should currently be interpreted primarily as synaptic-level susceptibility rather than as proven greater metabolic demand of Ib/Ic somata, because direct subtype-comparative measurements of metabolic demand remain limited [26,27,68,137]. Noise exposure induces downregulation of the apoptosis-inducing factor (AIF) in type I SGNs and activates the AIF–CHCHD4 pathway, leading to mitochondrial dysfunction and subsequent degeneration of vulnerable SGN subtypes [26,80]. Importantly, repeated observations of Ib/Ic susceptibility across independent models suggest that mature subtype differences are not merely descriptive, but functionally relevant in pathological settings.

Ototoxic drugs also damage SGNs in adulthood, although the pattern differs from that seen during development. In adult rodents, combined kanamycin and furosemide treatment typically causes rapid loss of sensory epithelium, followed by secondary SGN degeneration resulting from the loss of trophic support rather than immediate direct toxicity to SGN somata [93]. By contrast, gentamicin exposure alone can elevate auditory thresholds without obvious somatic loss of SGNs or OHCs in some models, suggesting that damage may occur primarily at afferent synapses or peripheral terminals rather than at the cell-body level [94]. Some reports further suggest that aminoglycosides can preferentially affect type I SGNs while sparing type II neurons [86], although this conclusion remains dependent on species, dose, and exposure paradigm. Thus, mature ototoxic injury appears to be subtype-sensitive, but also highly context-dependent.

Ischemic and hypoxic injuries provide a complementary perspective on subtype vulnerability. Type I SGNs are highly metabolically active and are therefore particularly sensitive to hypoxia [95]. Experimental work has shown that hypoxic stress can induce mitochondrial DNA damage, reduce ATP production, and promote apoptosis in type I SGNs [96,97,98]. Beyond subtype differences within type I SGNs, hypoxic injury also reveals marked divergence between type I and type II neurons. Type II SGNs, with their lower metabolic demands, can enhance the medial olivocochlear reflex via a neuroprotective pathway mediated by the purinergic receptor P2X3, thereby maintaining cellular homeostasis under hypoxia [99]. Hypoxia may be fatal for mature, highly differentiated SGNs [81]. However, cochlear spiral ganglion stem/progenitor cells (SGCs/SPCs) may show the opposite response, with increased stemness, proliferation, and glycolytic adaptation under hypoxia [100]. This contrast highlights an important principle: mature vulnerability is not simply determined by injury severity, but by the intrinsic biological state of the targeted cell population.

Additional systemic factors may also modulate mature SGN survival. Chronic lead exposure causes oxidative stress and accelerates degeneration by lowering glutathione and increasing malondialdehyde accumulation [106]. Nutritional imbalance may act through multiple pathways, including increased oxidative stress [107,108], micronutrient deficiency [109], inflammation secondary to recurrent middle-ear disease, and impaired cochlear microvascular function [110,111]. Although these factors are less clearly subtype-resolved than noise models, they further support the idea that mature SGN vulnerability is shaped by the interaction between cellular stress load and the metabolic or vascular context in which neurons operate.

Overall, mature-injury models support a view of SGN vulnerability as context-dependent rather than fixed. The same subtype may show different degrees of susceptibility across paradigms, but the repeated involvement of Ib/Ic-related populations suggests that intrinsic features, particularly metabolic demand, synaptic architecture, and stress-response capacity, meaningfully contribute to selective degeneration. This makes maturity the stage at which subtype identity becomes most visibly linked to pathological outcome.

### 4.3. Aging Stage: Cumulative Stress and Progressive Selective Degeneration

In aging, subtype-specific vulnerability appears to emerge not from acute developmental disruption or single-insult exposure but from the cumulative effects of chronic stress. Long-term mitochondrial dysfunction, oxidative imbalance, reduced trophic support, and chronic inflammation may progressively expose differences in subtype resilience, thereby promoting selective degeneration in the aging auditory nerve [81,138]. Aging therefore provides a particularly important test of whether subtype identity predicts long-term neural survival.

SGN degeneration is not an isolated peripheral event, because type I SGNs provide the first neural input from IHCs to the cochlear nucleus and thereby shape downstream auditory processing. Loss of IHC-SGN synapses or SGN fibers can reduce auditory nerve output, as reflected by reduced suprathreshold ABR wave I amplitudes, and may compromise temporal and intensity coding even when hearing thresholds are only modestly affected. Age-related hearing loss also involves central synapses of auditory nerve fibers and postsynaptic neurons in the cochlear nucleus, indicating that peripheral neural degeneration can be accompanied by broader auditory-circuit dysfunction [82]. Recent work further suggests that Ia/Ib/Ic-related inputs converge onto principal neurons of the anteroventral cochlear nucleus, rather than forming completely segregated central processing streams [67]. Therefore, selective peripheral loss of particular SGN subtypes may alter the composition, balance, and dynamic range of central auditory input, contributing to impaired speech-in-noise perception, reduced suprathreshold coding, and poorer rehabilitation outcomes.

Across both aging- and noise-related paradigms, type Ic SGNs have repeatedly been reported to decline earlier than other subtypes, supporting the hypothesis that their intrinsic molecular identity confers reduced long-term resilience [27]. In contrast, type Ia SGNs appear relatively resistant, possibly because of larger PSDs and more robust synaptic architecture [27,68]. Spatially, aging appears to interact with tonotopic organization, but the current evidence should be interpreted cautiously. Existing data support preferential age-related reduction in Ic-related neurons in middle and basal cochlear regions, rather than a simple conclusion that the basal cochlea is uniformly more vulnerable across all conditions [27]. These observations are consistent with the idea that subtype identity and cochlear location jointly determine vulnerability during aging.

Mitochondrial dysfunction is a central mechanism in this process. For example, mice lacking p43, a mitochondrial thyroid hormone receptor, show metabolic dysregulation, increased autophagy, abnormal myelination, and accelerated SGN degeneration accompanied by early severe age-related hearing loss (ARHL) [139]. Aging is also associated with reduced antioxidant capacity and accumulation of reactive oxygen species (ROS), which can directly damage neuronal structure and amplify glutamate-mediated excitotoxicity at IHC-SGN synapses [140]. Similar oxidative mechanisms have been implicated in both noise- and drug-induced injury, suggesting that aging may not represent a separate process so much as a prolonged accumulation of stress pathways that are also activated by other insults. Aging also weakens trophic support. Reduced production of, or responsiveness to, neurotrophic factors such as BDNF and NT-3 may render SGNs increasingly susceptible to secondary injury [141,142,143,144]. This reduction in supportive signaling is likely especially relevant for already vulnerable subtypes, because chronic insufficiency may progressively shift the balance from functional impairment to irreversible neuronal loss.

Recent single-cell and single-nucleus transcriptomic studies have begun to define molecular signatures of cochlear aging with greater precision. In cynomolgus macaques, single-cell profiling identified the downregulation of SLC35F1 as a feature of cochlear aging and further showed that metformin can reduce hair-cell loss and neuronal aging markers, potentially through anti-inflammatory and hearing-related transcriptional effects [145]. In mice, cross-temporal single-cell analysis has shown that aging-related transcriptional signatures and pro-apoptotic programs can emerge as early as mid-adulthood [146]. More recent single-nucleus work has implicated chronic inflammation and disrupted RNA splicing in inner-ear aging and downstream SGN loss [147]. Although these studies do not yet provide a complete subtype-resolved causal map, they strengthen the view that aging-related SGN degeneration reflects the interaction of subtype identity with progressive metabolic, inflammatory, and transcriptomic instability.

Overall, aging-related SGN degeneration is best understood as the cumulative outcome of subtype identity, chronic stress exposure, and declining tissue support. This framework helps explain why Ic-related populations repeatedly emerge as vulnerable in age-related auditory decline. It also suggests that successful intervention in ARHL may require not only general neuroprotection, but subtype-informed strategies that address mitochondrial maintenance, trophic support, and chronic inflammatory burden over time.

## 5. From Selective Vulnerability to Therapeutic Opportunity

**Figure 2:**
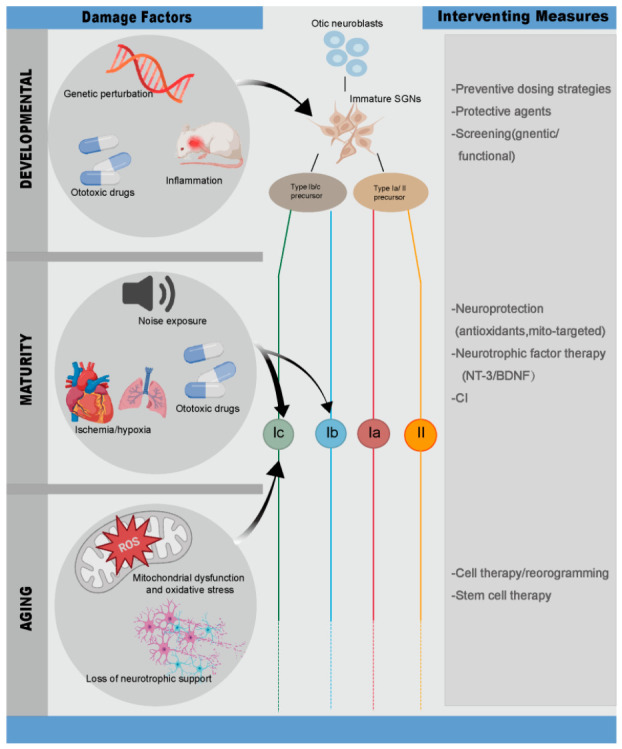
Lifespan-dependent vulnerability of spiral ganglion neuron subtypes and corresponding therapeutic strategies. Conceptual summary of the major factors associated with SGN injury across development, maturity, and aging, highlighting the SGN populations most likely to be affected at each life stage. During development, genetic disruption, ototoxic exposure, and inflammation may interfere with subtype specification and synaptogenesis. In the mature cochlea, subtype-specific physiological and metabolic properties shape injury responses to noise, ototoxic drugs, and ischemic stress. During aging, cumulative oxidative, mitochondrial, inflammatory, and trophic stress promotes progressive degeneration of vulnerable SGN populations. Representative intervention strategies aligned with different stages of SGN injury, including prevention, neuroprotection, trophic support, biomaterial-assisted repair, cochlear implant optimization, cell-based therapy, and identity reprogramming. Created in BioRender.

If SGN degeneration is shaped by the interaction between subtype identity, developmental stage, and injury context, then therapeutic strategies should not treat SGNs as a homogeneous neuronal population. Instead, effective intervention may require stage-aware and subtype-informed approaches that combine prevention, neuroprotection, repair, and functional restoration (Figure 2). The translational challenge is therefore not merely to preserve SGNs in general, but to identify which neuronal populations are most at risk, when intervention is most likely to succeed, and which mechanistic pathways are most actionable in each context. However, this framework should be interpreted as a preclinical and mechanistic roadmap rather than an immediately deployable clinical strategy, because most subtype-resolved evidence still derives from rodent models and lacks validated human biomarkers.

### 5.1. Prevention and Neuroprotection

Preventive and neuroprotective strategies aim to delay or avoid SGN injury before degeneration becomes irreversible. The most direct measures remain reduction in environmental risk, particularly avoidance of excessive noise and appropriate use of hearing protection devices [148]. However, because many forms of SGN injury converge on shared intracellular stress pathways, increasing attention has shifted toward mechanism-based neuroprotection.

Oxidative stress is one of the most consistently implicated pathogenic processes across SGN injury models. Antioxidant strategies, including N-acetylcysteine and shikonin, have shown partial protective effects in experimental systems [129,149]. Likewise, bisphosphonate treatment has been reported to increase synaptic counts after acoustic overexposure, suggesting possible benefit at the level of cochlear synapses rather than only neuronal somata [150]. Nutritional support may also contribute indirectly by helping preserve cochlear homeostasis and vascular integrity [112,116]. In ischemia-hypoxia-related injury models, studies indicate that mito-TEMPO exerts protective effects by preserving TFAM-mtDNA interactions [97] or enhancing endogenous neural stem cell activity [151,152].

For clinically indispensable but ototoxic agents such as cisplatin, neuroprotection must also include monitoring and risk management. Regular auditory surveillance and individualized assessment remain essential for early detection of injury and timely treatment adjustment. More recently, attention has also turned to regulated cell-death pathways, including ferroptosis, necroptosis, and pyroptosis, as possible therapeutic targets [38]. These pathways are attractive because they may offer a more mechanistically precise route to protection than broad antioxidant supplementation alone.

Nevertheless, most currently available neuroprotective approaches remain insufficiently subtype-resolved. They may delay degeneration or reduce general stress burden, but they do not yet selectively target the neuronal populations most at risk. This is a major translational limitation, because the cells most relevant to speech-in-noise performance, aging-related decline, or synaptopathy may not be the same across disease contexts. Thus, future neuroprotection will likely need to move beyond generalized cochlear preservation toward more explicitly subtype-informed intervention (Table 2). However, such interventions remain preclinical and cannot yet be considered clinically subtype-targeted.

### 5.2. Repair and Functional Enhancement

Once SGN injury occurs, the therapeutic goal shifts from prevention to repair, replacement, and functional enhancement of the auditory pathway. At this stage, the number and quality of surviving SGNs become key determinants of the ceiling for hearing rehabilitation. Cochlear implantation remains the most established clinical strategy for severe to profound hearing loss [43,156], but its efficacy depends strongly on the survival and physiological competence of residual SGNs.

This dependence is especially relevant in light of recent single-cell and subtype-resolved studies, which indicate that SGN loss is often selective rather than uniform [27,82]. Structural degeneration, altered neurite growth, and subtype imbalance may all reduce the precision with which electrical stimulation can encode complex acoustic information [163,164]. This may help explain why some CI users, particularly older adults, show poorer performance in speech recognition under noisy conditions [165]. These observations suggest that the future of SGN-directed therapy lies not only in stimulating residual neurons, but in improving the quality, organization, and responsiveness of the surviving auditory nerve.

Several approaches support this direction. Local round-window delivery of neurotrophin-3 (NT3) has been shown to promote synaptic repair after noise exposure [136,153], highlighting the value of bypassing the blood–labyrinth barrier and directly targeting vulnerable neural compartments. Cell-based strategies using mesenchymal stem cell-derived small extracellular vesicles or mesenchymal stem cells have also shown encouraging effects on SGN survival, neurite outgrowth, and functional recovery in experimental deafness models [166,167,168,169]. These approaches are attractive because they may provide trophic support even when direct neuronal replacement remains incomplete.

Biomaterial-based repair platforms extend this concept further. Biomimetic scaffolds can function both as delivery systems and as structural guides for directional neurite regeneration [156,157,158]. Hydrogels combined with neurotrophic factors such as BDNF can mimic extracellular matrix properties, prolong factor availability, and support SGN maintenance [159,160,161]. In deafened animal models, conductive and biodegradable scaffolds have also improved the spatial organization of the neurite-electrode interface [162], suggesting that engineering the microenvironment may meaningfully enhance CI performance and neural integration.

In sum, these repair-oriented strategies indicate that post-injury therapy should not be limited to preserving neurons in place. Rather, the broader goal should be restoration of auditory nerve quality. Among currently plausible early translational candidates, local neurotrophin delivery is the most directly connected to synaptic repair. NT-3 or BDNF delivered through the round window or intratympanic route may bypass systemic barriers and target the cochlear neural compartment more directly. Mitochondria-targeted antioxidants may be relevant where energetic stress and oxidative injury dominate, although subtype selectivity remains unproven. AAV-mediated neurotrophin delivery has shown benefit in larger-animal deafness models, supporting the feasibility of longer-lasting trophic support. In parallel, cochlear implant optimization, including improved electrode-neuron interfaces and stimulation strategies, may enhance the functional use of residual SGNs even when true subtype-specific rescue is not yet achievable.

### 5.3. Cell Replacement and Identity Reprogramming

Cell replacement strategies seek to restore the damaged auditory pathway by generating new SGN-like or inner-ear-supporting cells from stem-cell sources. Preclinical work has demonstrated that mesenchymal stem cells, embryonic stem cells, and induced pluripotent stem cells can be differentiated toward inner-ear progenitor or SGN-like states [9,154,155]. These approaches offer two potential benefits: replacement of lost cells and provision of trophic support to remaining neural elements.

Despite this promise, true functional replacement remains challenging. Newly generated cells must survive within the cochlear environment, adopt the appropriate neuronal identity, establish accurate peripheral and central connections, and integrate electrophysiologically into existing circuits. These requirements are especially important in the context of SGN subtype heterogeneity, because replacing neurons without restoring subtype-appropriate properties may not fully recover auditory coding.

This challenge makes identity reprogramming particularly interesting. Rather than replacing lost neurons de novo, reprogramming strategies attempt to alter the molecular identity of existing SGNs so that more vulnerable populations acquire properties associated with greater resilience. Identity reprogramming is attractive, but remains a preclinical proof-of-principle. For example, conditional Runx1 loss in mice can shift Ib/Ic SGNs toward more Ia-like molecular and functional features and enhance suprathreshold auditory nerve responses [72].

Although identity reprogramming is attractive, it remains a preclinical concept rather than a validated therapeutic modality. Most evidence comes from rodent systems, and important questions remain regarding safety, scalability, timing, and long-term functional integration in larger mammals. Even so, cell replacement and reprogramming studies are valuable not only because they suggest possible future therapies, but because they test the central hypothesis of this review that subtype identity is a meaningful and manipulable determinant of SGN vulnerability.

### 5.4. From Mouse to Human: Limitations and Translational Gaps

Subtype-informed frameworks for SGN vulnerability and intervention face several translational gaps when applied to human sensorineural hearing loss. First, the evidence base derives largely from rodent models and controlled injury paradigms. Most experimental studies use acute acoustic overexposure, ototoxic challenge, or defined genetic perturbations, whereas human hearing loss typically results from chronic, cumulative, and heterogeneous exposures over decades. Although mouse studies show that acoustic trauma and aging can differentially affect SGN subtypes [27,170,171,172], these paradigms do not capture the temporal complexity, comorbidities, and exposure histories of human disease. Primate aging data further indicate that age-related cochlear degeneration involves hair cells, SGNs, inflammation, and strial changes, highlighting the need to distinguish conserved mechanisms from rodent-specific patterns [145].

Second, cross-species differences constrain translation. Rodent and human cochleae differ in lifespan, cochlear size, tonotopic scaling, frequency range, and access to direct histopathological assessment. Recent primate studies suggest that early SGN subtype specification is conserved, whereas later maturation and mature marker expression show species-specific difference [66]. Therefore, molecular markers used to define mouse Ia/Ib/Ic SGNs should be regarded as candidate markers rather than definitive human subtype markers.

Third, much of the available evidence remains associative. Subtype identity, cochlear location, synaptic architecture, spontaneous rate, and injury outcome are linked by convergent evidence, but causal relationships have been directly tested in only a limited number of perturbation studies. In living humans, cochlear synaptopathy or SGN subtype loss cannot be quantified by routine audiometry, and candidate non-invasive biomarkers remain incompletely validated [173,174]. This complicates the interpretation of clinical endpoints such as speech-in-noise perception, tinnitus, hyperacusis, and hidden hearing loss, where human findings are heterogeneous and may reflect both peripheral and central mechanisms [173,174,175].

Finally, clinical implementation of subtype-targeted therapy requires solutions that are not yet established, including safe and spatially controlled local delivery, sustained therapeutic exposure, cell-type-specific targeting, appropriate timing, long-term safety monitoring, and outcome measures sensitive to suprathreshold function beyond threshold elevation. Advances in middle- and inner-ear drug delivery, including hydrogels, nanoparticles, microfluidic systems, and device-based approaches, provide relevant technical directions for overcoming cochlear delivery barriers, but current platforms do not yet establish subtype-selective SGN targeting in humans [176].

Together, these limitations indicate that subtype-informed SGN therapy remains a translational research direction rather than an established clinical strategy. While a subtype-resolved framework provides a useful basis for understanding SGN vulnerability, its clinical application will require human-relevant subtype validation, stronger causal evidence, improved cochlear delivery strategies, and functional outcome measures beyond threshold-based hearing tests. Thus, these gaps do not diminish the framework’s value but define the key steps needed before subtype-targeted approaches can be applied to human sensorineural hearing loss.

## 6. Conclusions and Future Perspectives

The evidence reviewed here supports the view that SGN degeneration in SNHL is not a uniform process. Rather, selective vulnerability emerges from the interaction between intrinsic subtype identity, life stage, and injury context. Advances in single-cell transcriptomics, molecular profiling, and functional analysis have moved the field beyond the traditional type I/type II framework and provided a more precise basis for understanding why different SGN populations may differ in resilience across the lifespan.

A lifespan perspective helps clarify how this vulnerability unfolds. During development, insults may disrupt subtype specification and synaptogenesis rather than simply cause generalized neuronal loss. In maturity, subtype-specific physiological and metabolic properties become more stable and appear to influence responses to noise, ototoxic exposure, and ischemic stress. During aging, cumulative mitochondrial dysfunction, oxidative imbalance, chronic inflammation, and declining trophic support progressively reveal differences in long-term resilience, with Ic-related populations repeatedly implicated as especially vulnerable.

This framework also has important translational implications. If the most clinically relevant forms of SGN degeneration are subtype-selective, then future therapies should move beyond generalized cochlear preservation. Stage-aware neuroprotection, subtype-informed repair, and more precise reprogramming or replacement strategies may ultimately provide greater benefit than approaches that treat all SGNs as biologically equivalent. At present, most candidate therapies remain preclinical, but the conceptual shift toward selective vulnerability already provides a stronger rationale for therapeutic prioritization.

A practical roadmap for translation should proceed through several sequential steps. First, SGN subtype identities and marker combinations must be validated across species, including rodents, non-human primates, and human cochlear tissue or human iPSC-derived inner-ear models. Comparative work in common marmoset and bat cochlea already suggests that some subtype-related programs are conserved, whereas other molecular features may be species-specific [66,76]; therefore, mouse Ia/Ib/Ic markers should not be assumed to map directly onto human SGNs without validation. Second, causal perturbation studies are needed to determine whether subtype identity itself drives vulnerability or merely correlates with injury outcome. Runx1 manipulation provides an important example that SGN subtype identity can be experimentally shifted [72], but additional perturbation studies targeting mitochondrial stress, synaptic architecture, neurotrophic dependence, and inflammatory pathways are needed. Third, clinically useful biomarkers must be developed to detect neural or subtype-biased injury before irreversible SGN loss occurs. Candidate approaches may include ABR wave I amplitude, extended high-frequency audiometry, and molecular biomarkers, but none currently provides a validated subtype-specific diagnostic measure in humans. Fourth, early-phase therapeutic candidates should be prioritized according to feasibility and mechanism, including local NT-3 or BDNF delivery [153], mitochondria-targeted antioxidants, neurotrophin gene therapy, and cochlear implant strategies that improve the interface with surviving SGNs. Crucially, intervention studies should be designed to assess not only overall SGN survival, but whether the most vulnerable and functionally consequential subtypes are specifically protected or restored.

In this sense, subtype identity should be viewed not merely as a descriptive feature of auditory neurons, but as an organizing principle for both mechanism and therapy. A review framework centered on selective vulnerability may therefore help connect auditory neuroscience, degeneration biology, and translational hearing research in a way that is both conceptually clearer and more clinically actionable.

## Figures and Tables

**Table 1:** Stage-dependent and subtype-specific vulnerability patterns of spiral ganglion neurons under major injury conditions.

Damage Category	Representative Insult(s)	Most Affected SGN Subtype(s)/Stage	Main Evidence and Differential Response	Proposed Mechanisms	**Functional/Clinical** **Relevance**	**References**
Noise	Acute or chronic noise exposure	Ic/Ib-associated type I SGNs	Ic-associated synapses are frequently reported to show the earliest and most prominent loss. Ia fibers exhibit the greatest resilience, with PSDs remaining stable or even enlarging compensatorily.	Plausible energetic or mitochondrial stress; direct subtype-comparative metabolic measurements remain limited. Mitochondrial vulnerability, including impairment of ATP synthesis pathways.	Reduced temporal precision and impaired coding in challenging listening conditions.	[24,25,26,77,78,79,80]
Age-related degeneration	Natural aging	Ic- or LSR-related neurons/synapses, especially in middle-to-basal regions in some models.	Synaptic loss may precede overt neuronal loss. Basal-turn Ic-related SGNs appear particularly vulnerable in several models. In human temporal bone studies, smaller neurons are reported to decline more rapidly, especially after 60 years of age.	Intrinsic physiological properties may contribute to reduced resilience during aging.	Presbycusis and poorer speech perception in noise.	[27,68,81,82]
Ototoxic drugs	Aminoglycosides (e.g., gentamicin, kanamycin)	Immature/early developmental SGNs	Developmental window: peak apoptosis around P7; SGNs highly vulnerable. Direct toxicity: kanamycin damages neonatal SGNs.	Oxidative stress. Activation of apoptotic pathways.	Developmental hearing loss.	[83,84,85,86,87,88,89]
	Platinum-based drugs (cisplatin)	Immature/developing SGNs	Younger age is consistently associated with higher clinical risk of cisplatin ototoxicity. Developing SGNs may be more susceptible to cisplatin-related injury.	Ferroptosis and oxidative injury have been proposed.	High-frequency hearing loss in pediatric cancer survivors.	[12,90,91,92]
	Aminoglycosides	Mature SGNs	Injury is often secondary to hair-cell loss rather than direct SGN toxicity. Gentamicin alone may not induce marked SGN morphological change in some mature models.	Reduced neurotrophic support after hair-cell degeneration.	Permanent SNHL with secondary neural degeneration.	[86,93,94]
Other factors	Ischemia/hypoxia	Type I > Type II; Mature SGNs > SPCs	Type I SGNs, including Ia/Ic-related populations in some studies, appear highly sensitive to ischemic stress. Mature SGNs are prone to excitotoxic injury. In contrast, SPCs may show enhanced stemness, proliferation, and glycolytic adaptation under hypoxia.	Oxidative stress, mitochondrial impairment. NMDAR overactivation. Metabolic reprogramming (glycolysis).	Hearing loss with possible activation of endogenous repair.	[81,95,96,97,98,99,100]
	Genetic defects	Ic or other subtype-specific SGNs	Hair-cell mutations may impair spontaneous activity and disrupt SGN subtype specification. Mitochondrial mutations directly increase the vulnerability of specific SGN subsets.	Disturbed developmental programs. Mitochondrial dysfunction.	Congenital or delayed-onset hearing loss.	[66,101,102,103,104,105]
	Environmental toxins & metabolic disturbances	Broad SGN involvement, with limited subtype resolution	Lead exposure triggers oxidative stress, causing SGN degeneration. Nutritional imbalance damages SGNs through oxidative stress, inflammation, and microvascular injury.	ROS accumulation. Spread of inflammatory mediators. Microvascular dysfunction.	Delayed or progressive hearing loss.	[106,107,108,109,110,111,112]

Inflammatory effects are discussed in the text but excluded from subtype comparison due to insufficient evidence.

**Table 2:** Emerging therapeutic strategies for spiral ganglion neuron injury and their potential relevance to subtype-selective vulnerability.

Intervention Strategy	Primary Therapeutic Aim	Potential Relevance to SGN Subtypes	Main Evidence and Advances	**References**
Pharmacological approaches	Reduce oxidative stress and inflammation, stabilize cochlear homeostasis, and delay SGN injury	Most current evidence concerns Type I SGNs, whereas true subtype selectivity remains limited	Antioxidants may mitigate injury induced by ototoxic drugs and noise exposure. Bisphosphonates have been reported to increase synaptic counts after acoustic overexposure. Nutritional support may help preserve a healthier cochlear microenvironment. Mitochondria-targeted agents may alleviate ischemia-hypoxia-related damage. In clinical practice, regular hearing monitoring and individualized risk assessment remain important for patients receiving ototoxic drugs.	[112,116,129,149]
Neurotrophic factors	Provide exogenous trophic support to promote neuronal survival, synaptic repair, and neurite outgrowth	Potential relevance to vulnerable Type I SGNs, particularly Ib/Ic-associated populations	Delivery through the round-window route has been reported to promote synaptic repair after noise exposure and may represent a promising strategy for cochlear synaptopathy.	[136,153]
Stem Cells and Cell Replacement	Replace damaged cells or provide trophic support for neural repair	Type I SGNs	Human induced pluripotent stem cells (hiPSCs) can be differentiated into SGN-like cells or used to generate inner-ear organoids. Mesenchymal stem cells, including dental pulp- and adipose-derived cells, can be induced toward neuronal phenotypes and have shown supportive effects on SGN survival and functional recovery after transplantation.	[9,154,155]
Biomaterials	Act as delivery vehicles, biomimetic scaffolds, or neural interfaces to guide regeneration and improve repair efficiency	Design-dependent, potential for subtype-specific targeting	Biomimetic scaffolds can function as targeted delivery platforms for bioactive factors. Their physicochemical properties may support directional neurite growth and functional recovery. As optimized electrode-neuron interfaces, such scaffolds may guide neurites toward electrodes and improve stimulation efficiency.	[156,157,158,159,160,161,162]
Cochlear Implant	Restore auditory input by electrically stimulating residual SGNs and combining stimulation with protective strategies	Primarily dependent on the number and functional integrity of residual Type I SGNs; subtype-informed optimization remains under investigation	Cochlear implants remain an effective approach for auditory rehabilitation, but outcomes depend on the number and function of residual SGNs. Current optimization strategies include combining implants with neurotrophic or protective agents and exploring novel stimulation modalities, such as optogenetics, for more precise neural encoding.	[43,156]

## Data Availability

No new data were created or analyzed in this study. Data sharing is not applicable to this article.

## References

[1] World Health Organization Deafness and Hearing Loss. World Health Organization. https://www.who.int/news-room/fact-sheets/detail/deafness-and-hearing-loss.

[2] Chadha S., Kamenov K., Cieza A. (2021). The world report on hearing, 2021. Bull. World Health Organ..

[3] Wilson B.S., Dorman M.F. (2008). Cochlear implants: A remarkable past and a brilliant future. Hear. Res..

[4] (2021). GBD 2019 Hearing Loss Collaborators. Hearing loss prevalence and years lived with disability, 1990–2019: Findings from the Global Burden of Disease Study 2019. Lancet.

[5] Tanna R.J., Lin J.W., De Jesus O. (2025). Sensorineural Hearing Loss. StatPearls.

[6] Liu Y., Qi J., Chen X., Tang M., Chu C., Zhu W., Li H., Tian C., Yang G., Zhong C. (2019). Critical role of spectrin in hearing development and deafness. Sci. Adv..

[7] Wang M., Han Y., Wang X., Liang S., Bo C., Zhang Z., Wang M., Xu L., Zhang D., Liu W. (2021). Characterization of EGR-1 Expression in the Auditory Cortex Following Kanamycin-Induced Hearing Loss in Mice. J. Mol. Neurosci..

[8] Wei H., Chen Z., Hu Y., Cao W., Ma X., Zhang C., Gao X., Qian X., Zhao Y., Chai R. (2021). Topographically Conductive Butterfly Wing Substrates for Directed Spiral Ganglion Neuron Growth. Small.

[9] Wang M., Xu L., Han Y., Wang X., Chen F., Lu J., Wang H., Liu W. (2021). Regulation of Spiral Ganglion Neuron Regeneration as a Therapeutic Strategy in Sensorineural Hearing Loss. Front. Mol. Neurosci..

[10] Boero L.E., Payne S., Gómez-Casati M.E., Rutherford M.A., Goutman J.D. (2021). Noise Exposure Potentiates Exocytosis From Cochlear Inner Hair Cells. Front. Synaptic Neurosci..

[11] Chen B., Sun Y., Sun H., Cong N., Ma R., Qian X., Lyu J., Fu X., Chi F., Li H. (2024). Ultrasound-Triggered NO Release to Promote Axonal Regeneration for Noise-Induced Hearing Loss Therapy. ACS Nano.

[12] Wang X., Xu L., Meng Y., Chen F., Zhuang J., Wang M., An W., Han Y., Chu B., Chai R. (2024). FOXO1-NCOA4 Axis Contributes to Cisplatin-Induced Cochlea Spiral Ganglion Neuron Ferroptosis via Ferritinophagy. Adv. Sci..

[13] Li J., Liu C., Müller U., Zhao B. (2023). Autophagy proteins are essential for aminoglycoside-induced hearing loss. Autophagy.

[14] Fetoni A.R., Paciello F., Troiani D. (2022). Cisplatin Chemotherapy and Cochlear Damage: Otoprotective and Chemosensitization Properties of Polyphenols. Antioxid. Redox Signal..

[15] Dang J., Bian P., Chen C., Chen C., Shan W., Cai L., Li Y., Tan H., Xu B., Guan M. (2025). Impact of POU3F4 mutation on cochlear development and auditory function. Cell Commun. Signal..

[16] Singh J., Randle M.R., Walters B.J., Cox B.C. (2024). The transcription factor Pou4f3 is essential for the survival of postnatal and adult mouse cochlear hair cells and normal hearing. Front. Cell. Neurosci..

[17] He Z.H., Li M., Fang Q.J., Liao F.L., Zou S.Y., Wu X., Sun H.Y., Zhao X.Y., Hu Y.J., Xu X.X. (2021). FOXG1 promotes aging inner ear hair cell survival through activation of the autophagy pathway. Autophagy.

[18] Kujawa S.G., Liberman M.C. (2015). Synaptopathy in the noise-exposed and aging cochlea: Primary neural degeneration in acquired sensorineural hearing loss. Hear. Res..

[19] Lou J., Wu F., Liu W., Hu R., He W., Feng Y., Huang Y., Guo J., Deng J., Zhao Z. (2025). Inhibition of TLR4 mitigates sensorineural hearing loss resulting from cochlear inflammation. Mol. Med..

[20] Zhang Y., Li Y., Fu X., Wang P., Wang Q., Meng W., Wang T., Yang J., Chai R. (2021). The Detrimental and Beneficial Functions of Macrophages After Cochlear Injury. Front. Cell Dev. Biol..

[21] Zhang L., Chen S., Sun Y. (2021). Mechanism and Prevention of Spiral Ganglion Neuron Degeneration in the Cochlea. Front. Cell. Neurosci..

[22] Liu C., Glowatzki E., Fuchs P.A. (2015). Unmyelinated type II afferent neurons report cochlear damage. Proc. Natl. Acad. Sci. USA.

[23] Wang L., Xu M., Zhang Q., Li G.L. (2024). Amitriptyline protects afferent synapses in the cochlea against excitotoxic trauma in vitro. FEBS J..

[24] Shrestha B.R., Chia C., Wu L., Kujawa S.G., Liberman M.C., Goodrich L.V. (2018). Sensory Neuron Diversity in the Inner Ear Is Shaped by Activity. Cell.

[25] Sun S., Babola T., Pregernig G., So K.S., Nguyen M., Su S.M., Palermo A.T., Bergles D.E., Burns J.C., Müller U. (2018). Hair Cell Mechanotransduction Regulates Spontaneous Activity and Spiral Ganglion Subtype Specification in the Auditory System. Cell.

[26] Franco J.A., Copeland T.G., Merrow R.D., Goodrich L.V. (2025). Molecularly defined auditory neuron subtypes show different vulnerabilities to noise- and age-related synaptopathy in mice. bioRxiv.

[27] Wang M., Lin S., Xie R. (2023). Apical-basal distribution of different subtypes of spiral ganglion neurons in the cochlea and the changes during aging. PLoS ONE.

[28] Anthwal N., Thompson H. (2016). The development of the mammalian outer and middle ear. J. Anat..

[29] Stauffer E.A., Holt J.R. (2007). Sensory transduction and adaptation in inner and outer hair cells of the mouse auditory system. J. Neurophysiol..

[30] Appler J.M., Goodrich L.V. (2011). Connecting the ear to the brain: Molecular mechanisms of auditory circuit assembly. Prog. Neurobiol..

[31] Raphael Y., Altschuler R.A. (2003). Structure and innervation of the cochlea. Brain Res. Bull..

[32] Kujawa S.G., Liberman M.C. (2019). Translating animal models to human therapeutics in noise-induced and age-related hearing loss. Hear. Res..

[33] Li L., Chao T., Brant J., O’Malley B., Tsourkas A., Li D. (2017). Advances in nano-based inner ear delivery systems for the treatment of sensorineural hearing loss. Adv. Drug Deliv. Rev..

[34] Zhang L., Chen X., Wang X., Zhou Y., Fang Y., Gu X., Zhang Z., Sun Q., Li N., Xu L. (2024). AAV-mediated Gene Cocktails Enhance Supporting Cell Reprogramming and Hair Cell Regeneration. Adv. Sci..

[35] Qi J., Huang W., Lu Y., Yang X., Zhou Y., Chen T., Wang X., Yu Y., Sun J.Q., Chai R. (2024). Stem Cell-Based Hair Cell Regeneration and Therapy in the Inner Ear. Neurosci. Bull..

[36] Li S., He S., Lu Y., Jia S., Liu Z. (2023). Epistatic genetic interactions between Insm1 and Ikzf2 during cochlear outer hair cell development. Cell Rep..

[37] Chen P., Hao J.J., Li M.W., Bai J., Guo Y.T., Liu Z., Shi P. (2022). Integrative Functional Transcriptomic Analyses Implicate Shared Molecular Circuits in Sensorineural Hearing Loss. Front. Cell Neurosci..

[38] Zhang S., Xiao H., Lin Y., Tang X., Tong W., Shao B., Li H., Xu L., Ding X., Chai R. (2025). Targeting Programmed Cell Death in Acquired Sensorineural Hearing Loss: Ferroptosis, Necroptosis, and Pyroptosis. Neurosci. Bull..

[39] Joshi Y., Savas J.N. (2025). A deafness-blindness syndrome results from ATF6-based disruption of the unfolded protein response. J. Clin. Invest..

[40] Qiu Y., Xie L., Wang X., Xu K., Bai X., Chen S., Sun Y. (2024). Abnormal Innervation, Demyelination, and Degeneration of Spiral Ganglion Neurons as Well as Disruption of Heminodes are Involved in the Onset of Deafness in Cx26 Null Mice. Neurosci. Bull..

[41] Wang X., Zhang M., Meng Y., An W., Wang M., Chen F., Chen L., Suo A., Xing Y., Kong L. (2025). An immortalized cochlear spiral ganglion neuronal cell line: A promising tool for hearing loss study. Neuroscience.

[42] Li Z., Gao Y., Chen X., Xu L., Li Z., Chai R. (2025). Study on Recovery Strategy of Hearing Loss & SGN Regeneration Under Physical Regulation. Adv. Sci..

[43] Nieratschker M., Yildiz E., Gerlitz M., Bera S., Gadenstaetter A.J., Kramer A.M., Kwiatkowska M., Mistrik P., Landegger L.D., Braun S. (2024). A preoperative dose of the pyridoindole AC102 improves the recovery of residual hearing in a gerbil animal model of cochlear implantation. Cell Death Dis..

[44] Seyyedi M., Viana L.M., Nadol J.B. (2014). Within-subject comparison of word recognition and spiral ganglion cell count in bilateral cochlear implant recipients. Otol. Neurotol..

[45] Matsuoka A.J., Morrissey Z.D., Zhang C., Homma K., Belmadani A., Miller C.A., Chadly D.M., Kobayashi S., Edelbrock A.N., Tanaka-Matakatsu M. (2017). Directed Differentiation of Human Embryonic Stem Cells Toward Placode-Derived Spiral Ganglion-like Sensory Neurons. Stem Cells Transl. Med..

[46] Qi J., Fu X., Zhang L., Tan F., Li N., Sun Q., Hu X., He Z., Xia M., Chai R. (2025). Current AAV-mediated gene therapy in sensorineural hearing loss. Fundam. Res..

[47] Zine A., Messat Y., Fritzsch B. (2021). A human induced pluripotent stem cell-based modular platform to challenge sensorineural hearing loss. Stem Cells.

[48] Andrade da Silva L.H., Heuer R.A., Roque C.B., McGuire T.L., Hosoya T., Kimura H., Tamura K., Matsuoka A.J. (2023). Enhanced survival of hypoimmunogenic otic progenitors following intracochlear xenotransplantation: Repercussions for stem cell therapy in hearing loss models. Stem Cell Res. Ther..

[49] Jia Y., Li H., Li W. (2025). Molecular identity of the mechanotransduction machinery in inner ear hair cells and mechanotransduction-linked hearing loss. Fundam. Res..

[50] Zhang K.D., Coate T.M. (2017). Recent advances in the development and function of type II spiral ganglion neurons in the mammalian inner ear. Semin. Cell Dev. Biol..

[51] Reijntjes D.O.J., Pyott S.J. (2016). The afferent signaling complex: Regulation of type I spiral ganglion neuron responses in the auditory periphery. Hear. Res..

[52] Whitlon D.S., Grover M., Tristano J., Williams T., Coulson M.T. (2007). Culture conditions determine the prevalence of bipolar and monopolar neurons in cultures of dissociated spiral ganglion. Neuroscience.

[53] Vyas P., Wu J.S., Jimenez A., Glowatzki E., Fuchs P.A. (2019). Characterization of transgenic mouse lines for labeling type I and type II afferent neurons in the cochlea. Sci. Rep..

[54] Bachman J.L., Kitcher S.R., Vattino L.G., Beaulac H.J., Chaves M.G., Hernandez Rivera I., Katz E., Wedemeyer C., Weisz C.J.C. (2025). GABAergic synapses between auditory efferent neurons and type II spiral ganglion afferent neurons in the mouse cochlea. Proc. Natl. Acad. Sci. USA.

[55] Smith F.L., Davis R.L. (2016). Organ of Corti explants direct tonotopically graded morphology of spiral ganglion neurons in vitro. J. Comp. Neurol..

[56] Petitpré C., Bourien J., Wu H., Diuba A., Puel J.-L., Lallemend F. (2020). Genetic and functional diversity of primary auditory afferents. Curr. Opin. Physiol..

[57] Moser T., Karagulyan N., Neef J., Jaime Tobón L.M. (2023). Diversity matters-extending sound intensity coding by inner hair cells via heterogeneous synapses. EMBO J..

[58] Siebald C., Vincent P.F.Y., Bottom R.T., Sun S., Reijntjes D.O.J., Manca M., Glowatzki E., Müller U. (2023). Molecular signatures define subtypes of auditory afferents with distinct peripheral projection patterns and physiological properties. Proc. Natl. Acad. Sci. USA.

[59] Karagulyan N., Überegger M., Qi Y., Babai N., Hofer F., Johnson Chacko L., Wang F., Luque M., Glueckert R., Schrott-Fischer A. (2025). Probing the role of synaptic adhesion molecule RTN4RL2 in setting up cochlear connectivity. eLife.

[60] Jaime Tobón L.M., Moser T. (2024). Bridging the gap between presynaptic hair cell function and neural sound encoding. eLife.

[61] Liberman M.C. (1978). Auditory-nerve response from cats raised in a low-noise chamber. J. Acoust. Soc. Am..

[62] Brown A.D., Benichoux V., Jones H.G., Anbuhl K.L., Tollin D.J. (2018). Spatial variation in signal and sensory precision both constrain auditory acuity at high frequencies. Hear. Res..

[63] Kujawa S.G., Liberman M.C. (2009). Adding insult to injury: Cochlear nerve degeneration after “temporary” noise-induced hearing loss. J. Neurosci..

[64] Furman A.C., Kujawa S.G., Liberman M.C. (2013). Noise-induced cochlear neuropathy is selective for fibers with low spontaneous rates. J. Neurophysiol..

[65] Petitpré C., Wu H., Sharma A., Tokarska A., Fontanet P., Wang Y., Helmbacher F., Yackle K., Silberberg G., Hadjab S. (2018). Neuronal heterogeneity and stereotyped connectivity in the auditory afferent system. Nat. Commun..

[66] Hosoya M., Ueno M., Shimanuki M.N., Nishiyama T., Oishi N., Ozawa H. (2024). A primate model animal revealed the inter-species differences and similarities in the subtype specifications of the spiral ganglion neurons. Sci. Rep..

[67] Wong N.F., Brongo S.E., Forero E.A., Sun S., Cook C.J., Lauer A.M., Müller U., Xu-Friedman M.A. (2025). Convergence of Type 1 Spiral Ganglion Neuron Subtypes onto Principal Neurons of the Anteroventral Cochlear Nucleus. J. Neurosci..

[68] Sun Y., Liu Z. (2023). Recent advances in molecular studies on cochlear development and regeneration. Curr. Opin. Neurobiol..

[69] Pearson L.J., Pinyon J.L., Cederholm J.M.E., von Jonquieres G., Bartlett F., Vázquez-Campos X., Delerue F., Ittner L.M., Housley G.D. (2025). Developmental differentiation of mouse inner ear neuron subpopulations resolved with a peripherin-promoter reporter within the Grm8 locus. Sci. Rep..

[70] Bastille I., Lee L., Moncada-Reid C., Yu W.M., Sitko A., Yung A., Zamani M., Christophersen N., Maroofian R., Galehdari H. (2025). Combinatorial transcriptional regulation establishes subtype-appropriate synaptic properties in auditory neurons. Cell Rep..

[71] Pai E.L., Vogt D., Clemente-Perez A., McKinsey G.L., Cho F.S., Hu J.S., Wimer M., Paul A., Fazel Darbandi S., Pla R. (2019). Mafb and c-Maf Have Prenatal Compensatory and Postnatal Antagonistic Roles in Cortical Interneuron Fate and Function. Cell Rep..

[72] Shrestha B.R., Wu L., Goodrich L.V. (2023). Runx1 controls auditory sensory neuron diversity in mice. Dev. Cell.

[73] Sherrill H.E., Jean P., Driver E.C., Sanders T.R., Fitzgerald T.S., Moser T., Kelley M.W. (2019). Pou4f1 Defines a Subgroup of Type I Spiral Ganglion Neurons and Is Necessary for Normal Inner Hair Cell Presynaptic Ca^2+^ Signaling. J. Neurosci..

[74] Liu Q., Lee E., Davis R.L. (2014). Heterogeneous intrinsic excitability of murine spiral ganglion neurons is determined by Kv1 and HCN channels. Neuroscience.

[75] Batrel C., Huet A., Hasselmann F., Wang J., Desmadryl G., Nouvian R., Puel J.L., Bourien J. (2017). Mass Potentials Recorded at the Round Window Enable the Detection of Low Spontaneous Rate Fibers in Gerbil Auditory Nerve. PLoS ONE.

[76] Bao M., Wang X., Li X., Sun R., Wang Z., Jiang T., Wang H., Feng J. (2025). Single-Cell Landscape of the Cochlea Revealed Cell-Type-Specific Diversification in Hipposideros armiger Based on PacBio Long-Read Sequencing. Biomolecules.

[77] Reijntjes D.O.J., Burke K., Paul S., Mueller U., Glowatzki E., Lauer A.M. (2026). Increased vulnerability to noise exposure of low spontaneous rate type 1C spiral ganglion neuron synapses with inner hair cells. Hear. Res..

[78] Stamataki S., Francis H.W., Lehar M., May B.J., Ryugo D.K. (2006). Synaptic alterations at inner hair cells precede spiral ganglion cell loss in aging C57BL/6J mice. Hear. Res..

[79] Guo L., Cao W., Niu Y., He S., Chai R., Yang J. (2021). Autophagy Regulates the Survival of Hair Cells and Spiral Ganglion Neurons in Cases of Noise, Ototoxic Drug, and Age-Induced Sensorineural Hearing Loss. Front. Cell. Neurosci..

[80] Ding Z.J., Wang Y., Wang R.F., Mi W.J., Qiu J.H., Zha D.J. (2022). Expression of complexes I, II and IV in the SGNs of noise-stimulated rats was decreased, and mitochondrial energy metabolism was disturbed, mediating the damage and degeneration of SGNs. bioRxiv.

[81] Kaur C., Saini S., Pal I., Kumar P., Chandra Sati H., Jacob T.G., Bhardwaj D.N., Roy T.S. (2020). Age-related changes in the number of cresyl-violet-stained, parvalbumin and NMDAR 2B expressing neurons in the human spiral ganglion. Hear. Res..

[82] Xie R., Wang M., Zhang C. (2024). Mechanisms of age-related hearing loss at the auditory nerve central synapses and postsynaptic neurons in the cochlear nucleus. Hear. Res..

[83] Zimmerman E., Lahav A. (2013). Ototoxicity in preterm infants: Effects of genetics, aminoglycosides, and loud environmental noise. J. Perinatol..

[84] Musiime G.M., Seale A.C., Moxon S.G., Lawn J.E. (2015). Risk of gentamicin toxicity in neonates treated for possible severe bacterial infection in low- and middle-income countries: Systematic Review. Trop. Med. Int. Health.

[85] Laurell G. (2019). Pharmacological intervention in the field of ototoxicity. HNO.

[86] Gao K., Ding D., Sun H., Roth J., Salvi R. (2017). Kanamycin Damages Early Postnatal, but Not Adult Spiral Ganglion Neurons. Neurotox. Res..

[87] Li H., Chai R. (2019). Hearing Loss: Mechanisms, Prevention and Cure.

[88] Hou S., Chen P., Chen J., Chen J., Sun L., Chen J., He B., Li Y., Qin H., Hong Y. (2020). Distinct Expression Patterns of Apoptosis and Autophagy-Associated Proteins and Genes during Postnatal Development of Spiral Ganglion Neurons in Rat. Neural Plast..

[89] Bai Y., Liu J., Wu X., Pang B., Zhang S., Jiang M., Chen A., Huang H., Chen Y., Zeng Y. (2023). Susceptibility of immature spiral ganglion neurons to aminoglycoside-induced ototoxicity is mediated by the TRPV1 channel in mice. Hear. Res..

[90] Lanvers-Kaminsky C., Zehnhoff-Dinnesen A.A., Parfitt R., Ciarimboli G. (2017). Drug-induced ototoxicity: Mechanisms, Pharmacogenetics, and protective strategies. Clin. Pharmacol. Ther..

[91] Olgun Y., Aktaş S., Altun Z., Kırkım G., Kızmazoğlu D., Erçetin A.P., Demir B., İnce D., Mutafoğlu K., Demirağ B. (2016). Analysis of genetic and non genetic risk factors for cisplatin ototoxicity in pediatric patients. Int. J. Pediatr. Otorhinolaryngol..

[92] Shahab M., Rosati R., Stemmer P.M., Dombkowski A., Jamesdaniel S. (2024). Quantitative profiling of cochlear synaptosomal proteins in cisplatin-induced synaptic dysfunction. Hear. Res..

[93] Ye B., Wang Q., Hu H., Shen Y., Fan C., Chen P., Ma Y., Wu H., Xiang M. (2019). Restoring autophagic flux attenuates cochlear spiral ganglion neuron degeneration by promoting TFEB nuclear translocation via inhibiting MTOR. Autophagy.

[94] Zhao N., Tai X., Zhai L., Shi L., Chen D., Yang B., Ji F., Hou K., Yang S., Gong S. (2017). Unitary ototoxic gentamicin exposure may not disrupt the function of cochlear outer hair cells in mice. Acta Otolaryngol..

[95] Ruan Q., Chen D., Wang Z., Chi F., He J., Wang J., Yin S. (2010). Effects of Kir2.1 gene transfection in cochlear hair cells and application of neurotrophic factors on survival and neurite growth of co-cultured cochlear spiral ganglion neurons. Mol. Cell. Neurosci..

[96] Lv P., Wei D., Yamoah E.N. (2010). Kv7-type channel currents in spiral ganglion neurons: Involvement in sensorineural hearing loss. J. Biol. Chem..

[97] Chen J.W., Ma P.W., Yuan H., Wang W.L., Lu P.H., Ding X.R., Lun Y.Q., Yang Q., Lu L.J. (2022). mito-TEMPO Attenuates Oxidative Stress and Mitochondrial Dysfunction in Noise-Induced Hearing Loss via Maintaining TFAM-mtDNA Interaction and Mitochondrial Biogenesis. Front. Cell. Neurosci..

[98] Yukawa H., Shen J., Harada N., Cho-Tamaoka H., Yamashita T. (2005). Acute effects of glucocorticoids on ATP-induced Ca2+ mobilization and nitric oxide production in cochlear spiral ganglion neurons. Neuroscience.

[99] Li Y., Dai P., Shen N., Huang X., Yin D. (2025). Role of purinergic receptors 2X3 on type II spiral ganglion neurons in the enhancement of medial olivocochlear reflex in mice after long-term noise exposure. Neuroreport.

[100] Chen H.C., Lee J.T., Shih C.P., Chao T.T., Sytwu H.K., Li S.L., Fang M.C., Chen H.K., Lin Y.C., Kuo C.Y. (2015). Hypoxia Induces a Metabolic Shift and Enhances the Stemness and Expansion of Cochlear Spiral Ganglion Stem/Progenitor Cells. BioMed Res. Int..

[101] Fu X., Wan P., Lu L., Wan Y., Liu Z., Hong G., Cao S., Bi X., Zhou J., Qiao R. (2023). Peroxisome Deficiency in Cochlear Hair Cells Causes Hearing Loss by Deregulating BK Channels. Adv. Sci..

[102] Feng Q., Jiang L., Zhang S., He C., Mei L., Liu Y. (2025). A novel frameshift mutation in the DIAPH1 gene causes a Chinese family autosomal dominant nonsyndromic hearing loss: Mutation in DIAPH1 causes hearing loss. Gene.

[103] Chen D.Y., Zhu W.D., Chai Y.C., Chen Y., Sun L., Yang T., Wu H. (2015). Mutation in PCDH15 may modify the phenotypic expression of the 7511T>C mutation in MT-TS1 in a Chinese Han family with maternally inherited nonsyndromic hearing loss. Int. J. Pediatr. Otorhinolaryngol..

[104] Seal R.P., Akil O., Yi E., Weber C.M., Grant L., Yoo J., Clause A., Kandler K., Noebels J.L., Glowatzki E. (2008). Sensorineural deafness and seizures in mice lacking vesicular glutamate transporter 3. Neuron.

[105] Chou C.W., Hsu Y.C. (2023). Current development of patient-specific induced pluripotent stem cells harbouring mitochondrial gene mutations and their applications in the treatment of sensorineural hearing loss. Hear. Res..

[106] Hu S.S., Cai S.Z., Kong X.Z. (2019). Chronic Lead Exposure Results in Auditory Deficits and Disruption of Hair Cells in Postweaning Rats. Oxid. Med. Cell. Longev..

[107] Jin D., Ge X., Liu L., Jia Y., Liu C., Meng K., Zhang X. (2025). Beyond Molecular Chaperoning: AHA1 Reprograms Autophagy Flux through Direct ATP5A1 Interaction in Ischemic Neuronal Injury. Redox Biol..

[108] Lorca C., Serra A., Gallart-Palau X. (2025). Mapping the oxidative stress metabolome in neurology by gas chromatography–mass spectrometry: A systematic review on signature-driven diagnosis and disease monitoring. Redox Biol..

[109] Maison S.F., Yin Y., Liberman L.D., Liberman M.C. (2016). Perinatal thiamine deficiency causes cochlear innervation abnormalities in mice. Hear. Res..

[110] Cosentino A., Agafonova A., Modafferi S., Trovato Salinaro A., Scuto M., Maiolino L., Fritsch T., Calabrese E.J., Lupo G., Anfuso C.D. (2024). Blood-Labyrinth Barrier in Health and Diseases: Effect of Hormetic Nutrients. Antioxid. Redox Signal..

[111] Neng L., Shi X. (2020). Vascular pathology and hearing disorders. Curr. Opin. Physiol..

[112] Jung S.Y., Kim S.H., Yeo S.G. (2019). Association of Nutritional Factors with Hearing Loss. Nutrients.

[113] Moore J.K., Linthicum F.H. (2007). The human auditory system: A timeline of development. Int. J. Audiol..

[114] Steinacher C., Nishio S.Y., Usami S.I., Dudas J., Rieder D., Rask-Andersen H., Crespo B., Moreno N., Konschake M., Seifarth C. (2024). Expression of Neurotrophins and Its Receptors During Fetal Development in the Human Cochlea. Int. J. Mol. Sci..

[115] Niknazar S., Abbaszadeh H.A., Khoshsirat S., Mehrjerdi F.Z., Peyvandi A.A. (2022). Combined treatment of retinoic acid with olfactory ensheathing cells protect gentamicin-induced SGNs damage in the rat cochlea in vitro. Mol. Cell. Neurosci..

[116] Zhang X., Zhou K., Tian K., Zhu Q., Liu W., Liu Z., An X., Tian C., Li Y., Lu F. (2022). VDR Regulates BNP Promoting Neurite Growth and Survival of Cochlear Spiral Ganglion Neurons through cGMP-PKG Signaling Pathway. Cells.

[117] Sun Y., Wang L., Zhu T., Wu B., Wang G., Luo Z., Li C., Wei W., Liu Z. (2022). Single-cell transcriptomic landscapes of the otic neuronal lineage at multiple early embryonic ages. Cell Rep..

[118] Petitpré C., Faure L., Uhl P., Fontanet P., Filova I., Pavlinkova G., Adameyko I., Hadjab S., Lallemend F. (2022). Single-cell RNA-sequencing analysis of the developing mouse inner ear identifies molecular logic of auditory neuron diversification. Nat. Commun..

[119] Sanders T.R., Kelley M.W. (2022). Specification of neuronal subtypes in the spiral ganglion begins prior to birth in the mouse. Proc. Natl. Acad. Sci. USA.

[120] Chen Y., Mu W., Wu Y., Xu J., Li X., Hu H., Wang S., Wang D., Hui B., Wang L. (2024). Optogenetically modified human embryonic stem cell-derived otic neurons establish functional synaptic connection with cochlear nuclei. J. Tissue Eng..

[121] Ng L., Goodyear R.J., Woods C.A., Schneider M.J., Diamond E., Richardson G.P., Kelley M.W., Germain D.L., Galton V.A., Forrest D. (2004). Hearing loss and retarded cochlear development in mice lacking type 2 iodothyronine deiodinase. Proc. Natl. Acad. Sci. USA.

[122] Xu Z., Tu S., Pass C., Zhang Y., Liu H., Diers J., Fu Y., He D.Z.Z., Zuo J. (2022). Profiling mouse cochlear cell maturation using 10× Genomics single-cell transcriptomics. Front. Cell. Neurosci..

[123] Economou A., Datta P., Georgiadis V., Cadot S., Frenz D., Maconochie M. (2013). Gata3 directly regulates early inner ear expression of Fgf10. Dev. Biol..

[124] Filova I., Bohuslavova R., Tavakoli M., Yamoah E.N., Fritzsch B., Pavlinkova G. (2022). Early Deletion of Neurod1 Alters Neuronal Lineage Potential and Diminishes Neurogenesis in the Inner Ear. Front. Cell Dev. Biol..

[125] Rahman M.T., Bailey E.M., Gansemer B.M., Pieper A.A., Manak J.R., Green S.H. (2023). Anti-inflammatory Therapy Protects Spiral Ganglion Neurons After Aminoglycoside Antibiotic-Induced Hair Cell Loss. Neurotherapeutics.

[126] Liu W., Xu L., Wang X., Zhang D., Sun G., Wang M., Wang M., Han Y., Chai R., Wang H. (2021). PRDX1 activates autophagy via the PTEN-AKT signaling pathway to protect against cisplatin-induced spiral ganglion neuron damage. Autophagy.

[127] Fu X., Wan P., Li P., Wang J., Guo S., Zhang Y., An Y., Ye C., Liu Z., Gao J. (2021). Mechanism and Prevention of Ototoxicity Induced by Aminoglycosides. Front. Cell. Neurosci..

[128] Nguyen T., Jeyakumar A. (2019). Genetic susceptibility to aminoglycoside ototoxicity. Int. J. Pediatr. Otorhinolaryngol..

[129] Du H., Zhou X., Shi L., Xia M., Wang Y., Guo N., Hu H., Zhang P., Yang H., Zhu F. (2022). Shikonin Attenuates Cochlear Spiral Ganglion Neuron Degeneration by Activating Nrf2-ARE Signaling Pathway. Front. Mol. Neurosci..

[130] Tserga E., Moreno-Paublete R., Sarlus H., Björn E., Guimaraes E., Göritz C., Cederroth C.R., Canlon B. (2020). Circadian vulnerability of cisplatin-induced ototoxicity in the cochlea. FASEB J..

[131] Gansemer B.M., Rahman M.T., Zhang Z., Green S.H. (2024). Spiral ganglion neuron degeneration in aminoglycoside-deafened rats involves innate and adaptive immune responses not requiring complement. Front. Mol. Neurosci..

[132] Wu C.C., Brugeaud A., Seist R., Lin H.C., Yeh W.H., Petrillo M., Coppola G., Edge A.S.B., Stankovic K.M. (2020). Altered expression of genes regulating inflammation and synaptogenesis during regrowth of afferent neurons to cochlear hair cells. PLoS ONE.

[133] Sung C.Y.W., Seleme M.C., Payne S., Jonjic S., Hirose K., Britt W. (2019). Virus-induced cochlear inflammation in newborn mice alters auditory function. JCI Insight.

[134] Lyu A.R., Kim S.J., Park M.J., Park Y.H. (2024). CORM-2 reduces cisplatin accumulation in the mouse inner ear and protects against cisplatin-induced ototoxicity. J. Adv. Res..

[135] Su Z., Liu Y., Zhang W., Liang W., Chen Y., Cao J., Liu Y., Zheng Y., Li Q. (2024). Endoplasmic reticulum stress-induced necroptosis promotes cochlear inflammation: Implications for age-related hearing loss. Exp. Gerontol..

[136] Liberman M.C., Kujawa S.G. (2017). Cochlear synaptopathy in acquired sensorineural hearing loss: Manifestations and mechanisms. Hear. Res..

[137] Wan G., Corfas G. (2015). No longer falling on deaf ears: Mechanisms of degeneration and regeneration of cochlear ribbon synapses. Hear. Res..

[138] Tan W.J.T., Song L. (2023). Role of mitochondrial dysfunction and oxidative stress in sensorineural hearing loss. Hear. Res..

[139] Affortit C., Casas F., Ladrech S., Ceccato J.C., Bourien J., Coyat C., Puel J.L., Lenoir M., Wang J. (2021). Exacerbated age-related hearing loss in mice lacking the p43 mitochondrial T3 receptor. BMC Biol..

[140] Saidia A.R., François F., Casas F., Mechaly I., Venteo S., Veechi J.T., Ruel J., Puel J.L., Wang J. (2024). Oxidative Stress Plays an Important Role in Glutamatergic Excitotoxicity-Induced Cochlear Synaptopathy: Implication for Therapeutic Molecules Screening. Antioxidants.

[141] Bardhan T., Jeng J.Y., Waldmann M., Ceriani F., Johnson S.L., Olt J., Rüttiger L., Marcotti W., Holley M.C. (2019). Gata3 is required for the functional maturation of inner hair cells and their innervation in the mouse cochlea. J. Physiol..

[142] Grandi F.C., De Tomasi L., Mustapha M. (2020). Single-Cell RNA Analysis of Type I Spiral Ganglion Neurons Reveals a Lmx1a Population in the Cochlea. Front. Mol. Neurosci..

[143] Provenzano M.J., Minner S.A., Zander K., Clark J.J., Kane C.J., Green S.H., Hansen M.R. (2011). p75(NTR) expression and nuclear localization of p75(NTR) intracellular domain in spiral ganglion Schwann cells following deafness correlate with cell proliferation. Mol. Cell. Neurosci..

[144] Yan W., Liu W., Qi J., Fang Q., Fan Z., Sun G., Han Y., Zhang D., Xu L., Wang M. (2018). A Three-Dimensional Culture System with Matrigel Promotes Purified Spiral Ganglion Neuron Survival and Function In Vitro. Mol. Neurobiol..

[145] Sun G., Fu X., Zheng Y., Hong G., Liu Z., Luo B., Lei J., Lv D., Chang M., Xiao Y. (2025). Single-cell profiling identifies hair cell SLC35F1 deficiency as a signature of primate cochlear aging. Nat. Aging.

[146] Sun G., Zheng Y., Fu X., Zhang W., Ren J., Ma S., Sun S., He X., Wang Q., Ji Z. (2023). Single-cell transcriptomic atlas of mouse cochlear aging. Protein Cell.

[147] Xia M., Zhang F., Ma J., Li Y., Jia G., Wu M., Lou Y., Liu Y., Li L., Li H. (2025). Single-nucleus profiling of mouse inner ear aging uncovers cell type heterogeneity and hair cell subtype-specific age-related signatures. Cell Rep..

[148] Wang T.C., Chang T.Y., Tyler R., Lin Y.J., Liang W.M., Shau Y.W., Lin W.Y., Chen Y.W., Lin C.D., Tsai M.H. (2020). Noise Induced Hearing Loss and Tinnitus-New Research Developments and Remaining Gaps in Disease Assessment, Treatment, and Prevention. Brain Sci..

[149] Bermúdez-Muñoz J.M., Celaya A.M., García-Mato Á., Muñoz-Espín D., Rodríguez-de la Rosa L., Serrano M., Varela-Nieto I. (2021). Dual-Specificity Phosphatase 1 (DUSP1) Has a Central Role in Redox Homeostasis and Inflammation in the Mouse Cochlea. Antioxidants.

[150] Seist R., Tong M., Landegger L.D., Vasilijic S., Hyakusoku H., Katsumi S., McKenna C.E., Edge A.S.B., Stankovic K.M. (2020). Regeneration of Cochlear Synapses by Systemic Administration of a Bisphosphonate. Front. Mol. Neurosci..

[151] Moon B.S., Ammothumkandy A., Zhang N., Peng L., Ibrayeva A., Bay M., Pratap A., Park H.J., Bonaguidi M.A., Lu W. (2018). The Presence of Neural Stem Cells and Changes in Stem Cell-like Activity with Age in Mouse Spiral Ganglion Cells In Vivo and In Vitro. Clin. Exp. Otorhinolaryngol..

[152] Song Z., Jadali A., Fritzsch B., Kwan K.Y. (2017). NEUROG1 Regulates CDK2 to Promote Proliferation in Otic Progenitors. Stem Cell Rep..

[153] Suzuki J., Corfas G., Liberman M.C. (2016). Round-window delivery of neurotrophin 3 regenerates cochlear synapses after acoustic overexposure. Sci. Rep..

[154] Li H., Roblin G., Liu H., Heller S. (2003). Generation of hair cells by stepwise differentiation of embryonic stem cells. Proc. Natl. Acad. Sci. USA.

[155] Kurihara S., Fujioka M., Hirabayashi M., Yoshida T., Hosoya M., Nagase M., Kato F., Ogawa K., Okano H., Kojima H. (2022). Otic Organoids Containing Spiral Ganglion Neuron-like Cells Derived from Human-induced Pluripotent Stem Cells as a Model of Drug-induced Neuropathy. Stem Cells Transl. Med..

[156] Liu L., Chen M., Zhang J., Li H., Li Z., Song J., Ma S., Wang Y., Lou X. (2024). Oriented polyaniline/poly-l-lactic acid/gelatin nanofiber scaffolds promote outgrowth of spiral ganglion neurons. J. Biomed. Mater. Res. A.

[157] Santi P.A., Johnson S.B. (2013). Decellularized ear tissues as scaffolds for stem cell differentiation. J. Assoc. Res. Otolaryngol..

[158] Mellott A.J., Shinogle H.E., Nelson-Brantley J.G., Detamore M.S., Staecker H. (2017). Exploiting decellularized cochleae as scaffolds for inner ear tissue engineering. Stem Cell Res. Ther..

[159] Wille I., Harre J., Oehmichen S., Lindemann M., Menzel H., Ehlert N., Lenarz T., Warnecke A., Behrens P. (2022). Development of Neuronal Guidance Fibers for Stimulating Electrodes: Basic Construction and Delivery of a Growth Factor. Front. Bioeng. Biotechnol..

[160] Pettingill L.N., Minter R.L., Shepherd R.K. (2008). Schwann cells genetically modified to express neurotrophins promote spiral ganglion neuron survival in vitro. Neuroscience.

[161] Chang H.T., Heuer R.A., Oleksijew A.M., Coots K.S., Roque C.B., Nella K.T., McGuire T.L., Matsuoka A.J. (2020). An engineered three-dimensional stem cell niche in the inner ear by applying a nanofibrillar cellulose hydrogel with a sustained-release neurotrophic factor delivery system. Acta Biomater..

[162] Wise A.K., Fallon J.B., Neil A.J., Pettingill L.N., Geaney M.S., Skinner S.J., Shepherd R.K. (2011). Combining cell-based therapies and neural prostheses to promote neural survival. Neurotherapeutics.

[163] Heffer L.F., Sly D.J., Fallon J.B., White M.W., Shepherd R.K., O’Leary S.J. (2010). Examining the auditory nerve fiber response to high rate cochlear implant stimulation: Chronic sensorineural hearing loss and facilitation. J. Neurophysiol..

[164] Wise A.K., Hume C.R., Flynn B.O., Jeelall Y.S., Suhr C.L., Sgro B.E., O’Leary S.J., Shepherd R.K., Richardson R.T. (2010). Effects of localized neurotrophin gene expression on spiral ganglion neuron resprouting in the deafened cochlea. Mol. Ther..

[165] Fogerty D., Ahlstrom J.B., Dubno J.R. (2023). Sentence recognition with modulation-filtered speech segments for younger and older adults: Effects of hearing impairment and cognition. J. Acoust. Soc. Am..

[166] Chen A., Qu J., You Y., Pan J., Scheper V., Lin Y., Tian X., Shu F., Luo Y., Tang J. (2024). Intratympanic injection of MSC-derived small extracellular vesicles protects spiral ganglion neurons from degeneration. Biomed. Pharmacother..

[167] You Y., Chen A., Qu J., Guo Y., Pan J., Yu T., Shu F., Tang J., Zhang H. (2025). MSC-sEV Promote Regeneration of Cochlear Spiral Ganglion Neurons and Myelin Sheaths in 3D Culture System. Neurosci. Bull..

[168] Leake P.A., Rebscher S.J., Dore C., Akil O. (2019). AAV-Mediated Neurotrophin Gene Therapy Promotes Improved Survival of Cochlear Spiral Ganglion Neurons in Neonatally Deafened Cats: Comparison of AAV2-hBDNF and AAV5-hGDNF. J. Assoc. Res. Otolaryngol..

[169] Miller J.M., Le Prell C.G., Prieskorn D.M., Wys N.L., Altschuler R.A. (2007). Delayed neurotrophin treatment following deafness rescues spiral ganglion cells from death and promotes regrowth of auditory nerve peripheral processes: Effects of brain-derived neurotrophic factor and fibroblast growth factor. J. Neurosci. Res..

[170] Milon B., Shulman E.D., So K.S., Cederroth C.R., Lipford E.L., Sperber M., Sellon J.B., Sarlus H., Pregernig G., Shuster B. (2021). A cell-type-specific atlas of the inner ear transcriptional response to acoustic trauma. Cell Rep..

[171] Hickman T.T., Hashimoto K., Liberman L.D., Liberman M.C. (2021). Cochlear Synaptic Degeneration and Regeneration After Noise: Effects of Age and Neuronal Subgroup. Front. Cell. Neurosci..

[172] Jean P., Wong Jun Tai F., Singh-Estivalet A., Lelli A., Scandola C., Megharba S., Schmutz S., Roux S., Mechaussier S., Sudres M. (2023). Single-cell transcriptomic profiling of the mouse cochlea: An atlas for targeted therapies. Proc. Natl. Acad. Sci. USA.

[173] Ripley S., Xia L., Zhang Z., Aiken S.J., Wang J. (2022). Animal-to-Human Translation Difficulties and Problems With Proposed Coding-in-Noise Deficits in Noise-Induced Synaptopathy and Hidden Hearing Loss. Front. Neurosci..

[174] Valderrama J.T., de la Torre A., McAlpine D. (2022). The hunt for hidden hearing loss in humans: From preclinical studies to effective interventions. Front. Neurosci..

[175] Bramhall N.F., McMillan G.P. (2024). Perceptual Consequences of Cochlear Deafferentation in Humans. Trends Hear..

[176] Delaney D.S., Liew L.J., Lye J., Atlas M.D., Wong E.Y.M. (2023). Overcoming barriers: A review on innovations in drug delivery to the middle and inner ear. Front. Pharmacol..

